# Closing the Gap between Bio-Based and Petroleum-Based Plastic through Bioengineering

**DOI:** 10.3390/microorganisms10122320

**Published:** 2022-11-23

**Authors:** Dina Al-Khairy, Weiqi Fu, Amnah Salem Alzahmi, Jean-Claude Twizere, Shady A. Amin, Kourosh Salehi-Ashtiani, Alexandra Mystikou

**Affiliations:** 1 Division of Science and Math, New York University Abu Dhabi, Abu Dhabi 129188, United Arab Emirates; 2 Department of Marine Science, Ocean College, Zhejiang University & Donghai Laboratory, Zhoushan 316021, China; 3 Center for Genomics and Systems Biology (CGSB), Institute Abu Dhabi, New York University Abu Dhabi, Abu Dhabi 129188, United Arab Emirates; 4 Laboratory of Viral Interactomes Networks, Unit of Molecular Biology of Diseases, Interdisciplinary Cluster for Applied Genoproteomics (GIGA Institute), University of Liège, 4000 Liège, Belgium

**Keywords:** bioplastics, biodegradable, bio-based plastic, PLA, PHA, PHB

## Abstract

Bioplastics, which are plastic materials produced from renewable bio-based feedstocks, have been investigated for their potential as an attractive alternative to petroleum-based plastics. Despite the harmful effects of plastic accumulation in the environment, bioplastic production is still underdeveloped. Recent advances in strain development, genome sequencing, and editing technologies have accelerated research efforts toward bioplastic production and helped to advance its goal of replacing conventional plastics. In this review, we highlight bioengineering approaches, new advancements, and related challenges in the bioproduction and biodegradation of plastics. We cover different types of polymers, including polylactic acid (PLA) and polyhydroxyalkanoates (PHAs and PHBs) produced by bacterial, microalgal, and plant species naturally as well as through genetic engineering. Moreover, we provide detailed information on pathways that produce PHAs and PHBs in bacteria. Lastly, we present the prospect of using large-scale genome engineering to enhance strains and develop microalgae as a sustainable production platform.

## 1. Introduction

Along with the global increase in human population, Earth is facing several challenges, including global warming, erosion and depletion of soil, and waste accumulation. Of these, the accumulation of plastic waste has coincided with the rise in population and plastic product consumption. The world produced 390.7 million tons of plastic in 2021 [1].

The United States Environmental Protection Agency (EPA) announced that 12% of the total 292.4 million tons of Municipal Solid Waste (MSW) generated in 2018 was plastic waste. From the total MSW generated that year, 146.1 million tons (MT) accumulated in landfills, out of which plastics comprised 18.46%. In addition, 69.1 million tons of the total MSW generated in the same year were recycled, of which only 4.47% were plastics [2].

The reason plastic waste accumulates is that petroleum-based plastics are primarily non-degradable and remain in the environment for many years. As a result, concerns about the harmful effects of plastic waste accumulation on the environment have increased. When considering the statistics outlined above, environmental contamination with plastic waste is a growing concern. With the continuation of the current plastic production rates and waste accumulation trends, landfills and environments such as soils and oceans will roughly accumulate about 12,000 MT of plastic waste by the year 2050 [3].

Pollution resulting from continuous waste accumulation in oceans and the negative effects this has had on marine life has raised awareness of ethical and social responsibilities towards marine life and the marine environment [4]. Plastic released into the marine environment is resistant to degradation and accumulates in nature, increasing dangers to human livelihood [5]. For example, fish and other sources of seafood consume microplastics, which are then consumed by humans [6,7]. Chemicals used in plastics, such as phthalates, are often detected in humans, altering the endocrine system [8,9].

Microplastics are plastic particles up to 5 mm in size that bioaccumulate in species such as fish, gastropods, crustaceans, marine mammals, and birds, as well as in drinking water, honey, sugar, salt, and marine sentiments [10,11]. Microplastic pollution is so pervasive that it is present in six of the most profound marine ecosystems on Earth at a record of 10,890 m below the sea’s surface, where they were found to be ingested by deep-sea amphipods [12]. Conventional polymers that are used in plastics, such as polyethylene (PE), polypropylene (PP), polystyrene (PS), polyamide (PA), polyester (PES), polyvinylchloride (PVC), and polyethylene terephthalate (PET), are mixed with chemical additives and residual monomers that have ecotoxicological effects on living organisms. When a living organism accumulates microplastic in its body, these chemical pollutants, in combination with other free-floating pollutants, can cause reproductive impairment, neurotoxicity, and oxidative stress [13], having a greater effect in larger predators due to food web biomagnification [14,15]. Ecosystems are strongly negatively affected when constituent organisms ingest plastic debris or become entangled in them [16]. Among 34 studied species of seabirds, 74% were recorded to ingest plastic [17]. Studies performed in 2015 have estimated that up to 12.7 million out of 275 million metric tons of plastic waste generated in 2010 entered the ocean within the same year [18].

**Figure 1:**
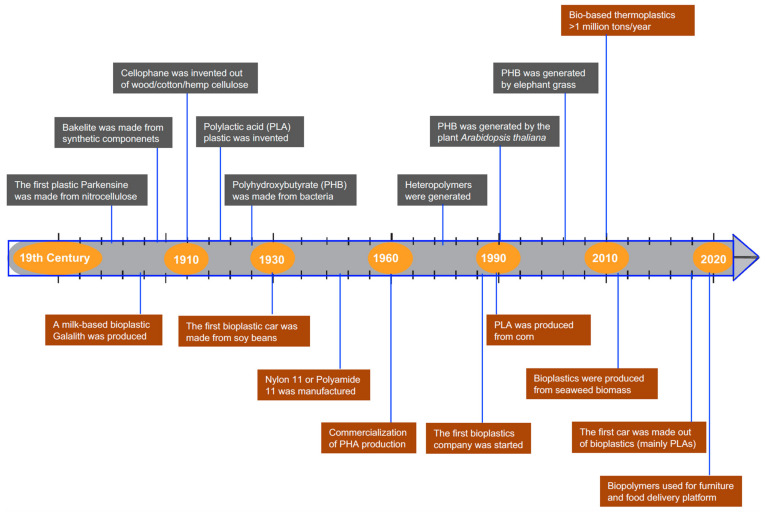
Timeline and milestones in bioplastic discovery and product development. Grey boxes indicate key innovations made in bioplastic research, and brown boxes show bioplastic production and applications in the context of technological advances.

It is evident that sustainable low-carbon societies require plastics derived from biomass instead of petroleum [19,20]. This can be achieved either with the use of polysaccharides derived from plants, with starch and cellulose being the most commonly used, or with microbial fermentation products, the most popular being polyhydroxyalkanoates (PHAs) and polylactic acid (PLA) [21,22]. Metabolic engineering allows native gene encoding for the formation of bioplastics in microbial organisms, such as *Cupriavidus necator* H16 (formerly *Ralstonia eutropha* H16), to be introduced through molecular genetics tools for optimal production of the desired compounds. One of the main disadvantages of using PHAs and PLA is that they have low thermal resistance, which limits their applications to packaging and dining supplies [23]. However, bioengineering enables the assembly of alternative microbial fermentation products with high thermal resistance when such properties are required [24]. Figure 1 summarizes the major milestones in inventions and commercialization in the bioplastics industry, showing that PHA, PHB, and PLA emerge as the main polymers [25].

**Figure 2:**
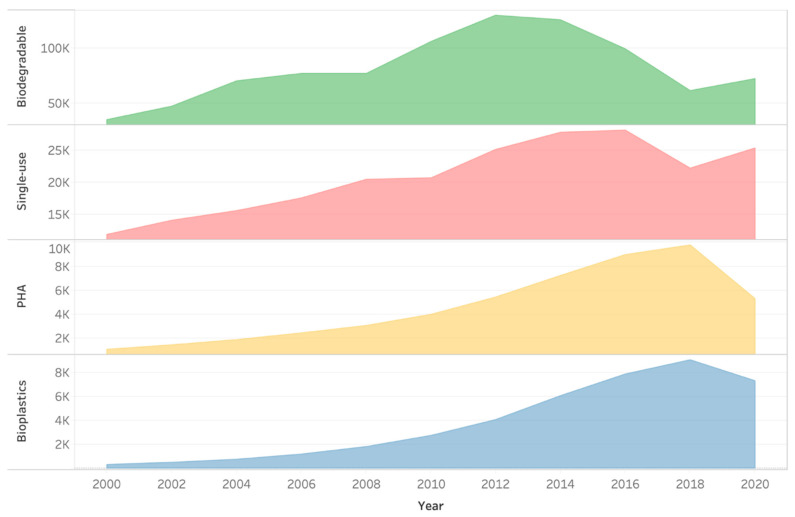
Google search engine indexed words related to the field in the last 20 years. The number of hits for single-use, bioplastics, biodegradable, and polyhydroxyalkanoates (PHA) are shown in two-year intervals from 2000 to 2020. The values for each searched word are shown in thousands (K) and are non-cumulative.

Bioplastics are produced from renewable biomass sources and can be biodegradable, thus are viable solutions to reduce non-degradable plastic waste, minimizing the hazards posed by petroleum-based plastics. Consequently, there has been more interest in this field in the last two decades, as evidenced by the rise in the number of studies on bioplastics. This increase likely reflects a growing concern manifested by research and scientific publications in the fields of bioplastics and biodegradability. Despite a decelerating period between 2018 and 2019, which may be related to the low crude oil prices during these years, bioplastics have gained noticeable interest in the last 20 years (Figure 2). Furthermore, in recent years, companies including Ikea and Nestle have started to use bioplastics in their products [26].

## 2. Native Polyhydroxyalkanoate Production in Microorganisms

PHAs are biodegradable polyester polymers that are used by many microbes as a means of storing energy. The native biosynthesis and accumulation of PHA occur in the cytoplasm of such microbial cells [27,28], where they are deposited within inclusion bodies in the form of granules ranging from 0.2 to 0.5 μm in diameter [29]. Such bacterial polyesters have properties that make them useful for applications such as thermoplastics, elastomers, and adhesives [30].

**Figure 3:**
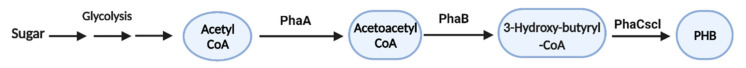
The PHB Biosynthesis Pathway *C. necator* H16. Condensation of 2 acetyl-CoA molecules by B-ketothiolase (PhaA), forming acetoacetyl-CoA, which is reduced by acetoacetyl-CoA reductase (PhaB). The last stage is polymerization by PHA synthase (PhaC).

PHAs differ in properties and chemical composition depending on the structural variation in the monomers constituting them [31]. Due to PHA polymers’ wide range of physical properties, such as flexibility, crystallinity, and thermal diversity [32], PHAs can be used as biodegradable plastics that can replace petroleum-based plastics. Environmental conditions and the type and composition of the PHA polymer affect its degradation speed, as different microorganisms produce different PHA-depolymerases to degrade PHAs [33]. For instance, poly-β hydroxybutyrate (PHB), the most popular PHA and the first member of the PHA family to be reported [34], is an energy storage compound produced by bacteria and microorganisms as an alternative energy storage molecule in the occasion of the scarcity of other nutrients [35]. The PHB biosynthesis pathway was the first to be characterized, and consists of three stages catalyzed by three enzymes in *C. necator* H16 [36]. The first stage is the condensation of two acetyl-CoA molecules, catalyzed by the first enzyme called B-ketothiolase (PhaA) forming acetoacetyl-CoA, which is reduced by the second enzyme acetoacetyl-CoA reductase (PhaB) [36]. The last stage is the polymerization by the third enzyme called polyhydroxyalkanoate synthase (PhaC) [35,37] (Figure 3).

## 3. Bioplastic Degradation

Not all bioplastics are biodegradable. A portion of polymers are made of renewable raw materials that are converted from biomass feedstock, while petroleum-based polymers derive from fossil fuels [38]. There is a misconception that all bioplastics are biodegradable due to the lack of valid information communicated to the public. For example, a survey took place in Australia in May of 2018 asking the public if they think that all bioplastics are biodegradable, with 21.9% responding that they agree with the statement, 70.4% responding that they are unsure, and only 7.7% responding that the statement is incorrect [39,40]. The policy (ISO 14855:1999) that defines if a bioplastic product is considered biodegradable requires it to degrade at least 90% naturally within six months, without leaving behind any toxic compounds [41]. Bioplastics can be non-biodegradable even if they derive from biobased polymers and biodegradable even if they derive from petroleum-based polymers [38]. More specifically, Table 1 presents the most commonly used and studied biobased and petroleum-based polymers with information about their biodegradability.

For instance, polylactic acid (PLA) is a compostable biodegradable that requires moderately high temperatures (45–50 °C) and moisture under aerobic conditions to biodegrade [42,43]. Thus, it requires composting since it is slow to biodegrade naturally [44]. While acetylcellulose’s (AcC) degradation rate depends on the degree of acetylation [45], and starch-based biodegradable plastics degrade naturally in soil, they are compostable under high temperatures (55–65 °C). Bioplastics may also contain a mixture of biobased and petroleum-based materials referred to as polymer blends that influence the degradation degree and performance of the bioplastic [45,46].

Every compostable, biodegradable polymer has different composting conditions and rates. Likewise, every naturally biodegradable polymer has a different rate of degradation that can be influenced by the climatic conditions of the environment [47]. In general, abiotic parameters such as exposure to ultraviolet (UV) irradiation, temperature, pH, oxygen, salinity, and chemicals in the environment influence the degradation of bioplastics in nature (soil and water) and in the composting environment [48,49]. There are four stages of biodegradation: biodeterioration, biofragmentation, bioassimilation, and mineralization. Biodeterioration is the initiation of the oxidation process by certain microorganisms. It is not clear if abiotic factors also influence this process, since sunlight or heat are usually present during microbial growth [49,50]. Microorganisms secrete enzymes that initiate biofragmentation and then bioassimilation of small fragments (<500 g/mol molar mass), in which the microbes catabolize the fragmented polymers for metabolism and growth [49,50]. Lastly, mineralization is the complete conversion of the carbon in the biopolymers to cell mass. We note that there is a gap in the literature regarding the complete conversion of the carbon in the polymers to cell mass, CO_2_, and H_2_0 [49,50].

**Table 1:** Source and biodegradability of commonly used bioplastics.

Abbreviation	Name	Fossil Fuel	Biomass	Non Biodegradable (NB)/ Biodegradable (B)	References
PHA	Poly(hydroxyalkanoate)		x	B	[43]
PHB	Poly(hyroxybutyrate)		x	B	[51]
PLA	Poly(lactide)		x	B	[44]
AcC (CTA, TAC)	Acetyl cellulose, cellulose triacetate		x	B	[45,52]
Starch	Starch		x	B	[46]
PBS	Poly(butylene succinate)	x		B	[45,51,53]
PES	Poly(ethylene succinate)	x		B	[45]
PCL	Poly(caprolactone)	x		B	[45,51]
Bio PE	Poly(ethylene)		x	NB	[54]
Bio PP	Poly(propylene)		x	NB	[54]

## 4. Bioplastic Degradation: Enzymes and Microorganisms

Bioplastics degrade faster in the presence of bacteria or fungi due to intracellular and extracellular enzymes [48]. These microorganisms were isolated from environments with documented bioplastic degradation activity and were identified and screened for candidate enzymes that are associated with the degradation of polymers used in bioplastics. The ones that showed associated enzymatic activity were studied for optimal conditions such as temperature, pH, and time required to degrade bioplastic polymers [43]. Each type of bioplastic requires its own degrader. Below are some examples of microorganisms and enzymes that have been identified as bioplastic degraders.

The bacteria *Pseudomonas aeruginosa* and *Bacillus subtilis* degrade PHA through enzymes and, potentially, by enhancing these enzymes through the secretion of surfactants [55]. PHB is broken down by multiple enzymes produced by the bacterial genus *Streptomyces* [56,57], including the species *Streptomyces ascomycinicus* that secretes the enzyme PHB depolymerase (PhaZ_Sa_), encoded by the *fkbU* gene, which has been expressed and tested for PHB degradation in other bacterial species [58]. Several strains of the bacterial genus *Amycolatopsis* and the fungus *Tritirachium album* degrade PLA after the secretion of a protease-type enzyme and Proteinase K, respectively [59]. In addition, species from the fungal genus *Aspergillus* can degrade starch via hydrolysis [46]. PBS and PES have a common degrader, a species from the bacterial genus *Leptothrix* [60]. Finally, the bacterial genera *Pseudomonas*, *Tenacibaculum*, *Alcanivorax* [51], and the fungal genera *Purpureocillium*, *Cladosporium* [61] include PCL degraders. Many bacterial and fungal organisms from diverse genera produce key enzymes that play a vital role in the bioplastics industry and define how sustainable a bioplastic polymer can be.

## 5. Bioengineering of Polyhydroxyalkanoate Production in Bacteria

The chemical processes involved in polymer production have fewer bottlenecks than microbial or enzymatic processes; however, pathway engineering mediated through synthetic biology can mediate competitive industrial bio-based products [62]. Various types of PHAs are produced naturally by numerous bacteria [63]. *C. necator* H16 is known to be a bacterial model organism for PHA production [64] and is generally utilized to produce bio-based and biodegradable plastics alternatives that are less harmful to the environment. PHA polymers are classified into three types: short-chain-length PHA (scl-PHA), medium-chain-length PHA (mcl-PHA), and long-chain-length PHA (lcl). Their classification depends on the number of carbon atoms in the PHA monomers that constitute the different polymer types. Scl-PHAs contain 3 to 5 carbon atoms in their monomer, mcl-PHAs contain 6 to 14 carbon atoms, and lcl-PHAs contain 15 or more carbon atoms [65]. The polymerization step of different types of PHA is catalyzed by PhaC synthases, which are classified into four classes depending on substrate specificity and the length of polymer they produce. Class I, Class III, and Class IV produce short-chain-length (scl) PHAs, while Class II produces medium-chain-length (mcl) PHAs [66]. Various domains in PHA synthases contain conserved amino acid residues that are important for their function. Mutations in these conserved amino acid positions can affect enzyme activity, stability, substrate specificity, and product length. For example, in a mutational study that focused on *P. aeruginosa* class II PHA synthase (PhaC), Amara and Rhem replaced the conserved Trp398 residue by phe and Ala residues respectively. Both mutations, Trp398Phe and Trp398Ala, led to inactivation of the enzyme, suggesting these residues are essential for enzyme activity [67].

PHA and PHB are produced naturally by several bacterial species, such as *C. necator* H16 *which* produces PHB, Poly(3-hydroxybutyrate-co-3-hydroxyvalerate) (PHBV), and Poly(3-hydroxybutyrate-co-4-hydroxybutyrate) (P3HB4HB) [68,69,70]; *Alcaligenes latus* which produces PHB; *Aeromonas hydrophila* which produces poly(3-hydroxybutyrate-co-3-hydroxyhexanoate) (PHBHHx); *Pseudomonas putida* and *Pseudomonas oleovorans* which produce medium-chain-length PHA (mcl PHA); and *Bacillus spp.* which produces PHB [68]. Knowing which polymer is produced by each species of bacteria can facilitate a boost in their production through genome engineering and provide more sustainable bioplastics solutions.

PHAs are also produced from genetically engineered bacterial species with inserted PHA-related protein-coding genes. The genetically modified PHA species include *Escherichia coli* that express PHB synthesis genes from in *C*. *necator*. These include genes that encode β-ketothiolase, acetoacetyl-CoA reductase and PHB synthase (*phbCAB*), and a gene that encodes *Vitreoscilla* hemoglobin (*vgb*) or just the former to produce PHB [68,71]; *C*. *necator* H16 with an *Aeromonas caviae* native gene that encodes PHA synthase (*phaC_Ac_*) to produce PHBHHx [68,72]; and *A*. *hydrophila* also with a *C*. *necator* H16 native gene that encodes for β-ketothiolase and acetoacetyl-CoA reductase (*phbAB*) and the *vgb* gene to produce PHBHHx [68,73].

PLA is the end product of polymerization of the biomonomer lactic acid through bacterial fermentation. The filamentous fungal species *Rhizopus arrhizus* and *Rhizopus oryzae* as well as many members of the bacterial genus *Lactobacilli* are among the *L*(+)-lactic acid-producing microbes [74,75]. D(−)-lactic acid (D-lactate) is a stereoisomer of L(+)-lactic acid that can be produced through genome engineering [74]. *E*. *coli* has been genetically engineered to overproduce D-lactate by the overexpression of the *ldhA* gene that encodes for D-lactate dehydrogenase. The disruption of either the gene *pflB*, which encodes for pyruvate-formate lyase, or the gene *pta,* which encodes for phosphoacetyltransferase in *E. coli*, is associated with the increased production of D-lactate [76]. PLA can also be produced through a one-step fermentation process, using the metabolic engineering of *E*. *coli* to produce the PLA homopolymer poly(3-hydroxybutyrate-lactate) and its copolymer P(3HB-co-LA). Disrupting the three genes that code for *acetate kinase* (*ackA*), *phosphoenolpyruvate carboxylase (ppc)*, and *acetaldehyde/alcohol dehydrogenase (adhE)* in *E. coli*, and replacing the promoter of the genes *ldhA* (D-lactate dehydrogenase) and *acs* (acetyl-CoA synthetase) with the trc promoter leads to overexpression of those two genes and results in the overproduction of D-lactate [77]. Likewise, a metabolic pathway that involves the introduction of the gene *Pct_Cp_* from the bacterium *Clostridium propionicum* was introduced into *E*. *coli* to produce PLA in vivo. *Pct_Cp_* encodes for propionate CoA transferases to produce lactyl-CoA. Lastly, the introduction of the *PhaC1_Ps6-19_* gene from a *Pseudomonas* species that encodes for PHA synthase 1 incorporates lactyl-CoA into the polymer [78].

## 6. Polyhydroxyalkanoate Production in Plants

While the potential of producing PHA in microbes has been investigated for decades, it still cannot compete with petroleum-derived plastics because the latter is more cost-effective [79]. The cost of producing conventional petro-chemical based plastics is 5–10 times lower, with the main factor being the cost of the carbon substrate [31,80]. Therefore, attention has been directed toward the production of PHA in plants. This is an excellent option, as agricultural infrastructure is well-developed and can be scaled up easily. Several plant species have been engineered to produce PHB in recent years. The availability of acetyl-CoA in plant cells makes the biosynthesis of PHA in transgenic plants possible, as it is the primary substrate in the PHB pathway. PHB is an example of a simple PHA that has properties equivalent to petroleum-based plastics. It can be produced in plants sustainably, and it can degrade in nature [81]. The attempts are widely spread throughout C3 plants such as the leaves of *Arabidopsis thaliana* and cytosol [82]. PHB levels of 0.1% of dry weight (DW) in *A. thaliana* have been obtained in the cytosol [83]. It has also been shown that PHA production in plastids of C3 plants improved tremendously compared to other plant parts. When production of PHB is carried out in chloroplasts, up to 14% of DW in *A. thaliana* has been obtained [84]. A later study resulted in higher levels of PHB, reaching up to 40% DW in *Arabidopsis* [85]. PHB has been produced in other agricultural species, such as rice, where PHB levels have reached 0.5% DW in the whole plant and in the cytosol [86]. In tobacco, a significant improvement has been achieved as researchers produced PHB from sterile plants, producing up to 1.7% DW obtained in the tissue culture cells. These numbers decreased when plants were transferred to soil [87]. Bohmert and colleagues demonstrated great success as PHB levels were obtained in stable transgenic fertile tobacco plants producing up to 18.8% DW and 8.8% DW in the leaves and whole plant, respectively [88]. In *Camelina sativa*, which is rich in oil content, scientists were able to produce high levels of PHB in the seeds; nevertheless, high levels led to impaired survival of the seedlings [81]. In a later study, Malik et al. reported the production of high levels of poly-3-hydroxybutyrate in plastids of Camelina sativa seeds by using seed-specific promoters and a plastid targeting signal on the N-terminus of the PHB biosynthetic enzymes, leading to PHB levels of up to 15% of the mature seed weight in single T1 seeds [25]. A great step towards the commercialization of PHB-producing switchgrass has been demonstrated in work carried out by Somleva and colleagues, where up to 3.72% and 1.23% dry weight have been reported in both leaf tissue and whole tillers, respectively [89].

## 7. Polyhydroxyalkanoate Production in Microalgae

Algae’s phototropism and simple nutrient requirement make them excellent candidates for bioplastics production. Algae-based plastics have been a recent trend in the bioplastics era compared to traditional methods of utilizing feedstocks of corn and potatoes [90]. Microalgae and cyanobacteria are being used as feedstocks for PHB production in bacteria, where the ability of cyanobacteria to grow in a variety of environments and their high yield of biomass enables them to be a renewable and less expensive feedstock for PHB production in bacteria.

Moreover, cyanobacteria produce biopolymers as storage compounds to alleviate nutrient limitations under certain environmental conditions. For example, PHB accumulates in cyanobacteria under nitrogen and phosphorus starvation [91]. *Synechocystis* sp. CCALA192 cultures grown under nitrogen depletion had an average biomass concentration of 1.0 g/L with a PHB content of 12.5% of dry cell weight [92]. In addition, the genetic modifications of metabolic pathways have increased the productivity of PHB from cyanobacteria. For example, *Synechocystis* sp. PCC6803 cells accumulate PHBs during nitrogen or phosphorus starvation. The overexpression of transcription factor *sigE* has led to an increase in PhaA, PhaB, PhaC, and PhaE protein levels, which increased PHB levels in the presence or absence of nitrogen starvation [93].

Diatoms are another algal group that can survive in diverse environmental conditions and provide 40% of primary productivity in the marine environment ranging from lakes to oceans [94]. Genetic engineering advances from transgene expression, gene delivery methods, and genome-editing technologies facilitated PHA production attempts in diatoms [95]. Such efforts enabled inducible gene expression in *Cylindrotheca fusiformis* [96], which may open the way towards more options for diatoms as potential PHA producers. Attempts to introduce a PHB biosynthetic pathway from PHB-producing *R. eutopha* into the diatom *Phaeodactylum tricornutum* have been successful. In this instance, a nitrogen reductase inducible promoter was used to control the expression of bioplastic-making enzymes [97]. The expression of the *R. eutopha* PHB encoding genes resulted in PHB levels of up to 10.6% of dry algal weight, where granule-like structures containing PHB accumulated in the cytosol of *Phaeodactylum tricornutum* [97]. *Chlamydomonas reinhardtii* is a unicellular green microalga and it has an endogenous *phaA* gene. An expression vector containing PHB encoding genes from *R. eutopha, phaB,* and *phaC* was used to transform *C. reinhardtii,* and transgenes were integrated into the algal genome. The algal species successfully produced PHB amounts ranging from 3.3–6 µg PHB per g of algal DW. PHB granules were also detected using gas chromatography-mass spectrometry (GC–MC) and transmission electron microscopy (TEM) [98]. With the recent advances and the accelerating research efforts using algal species, PHA production in algae is a promising prospect.

## 8. Synthetic Approaches and New Advancements

**Figure 4:**
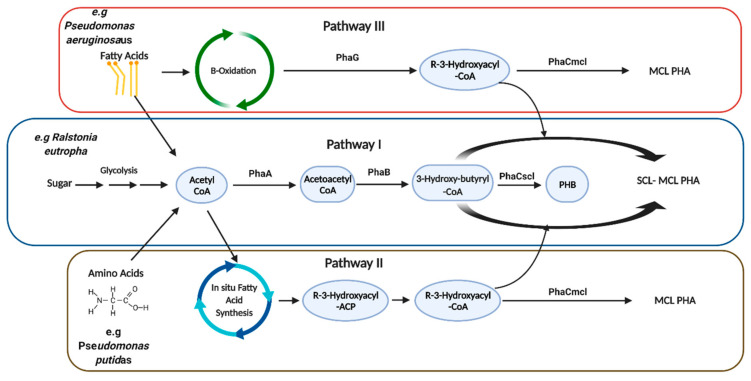
The Major PHA Biosynthesis Pathways. Acetoacetyl-CoA pathway (pathway I), in situ fatty acid synthesis (pathway II) and/or beta-oxidation cycles (pathway III). PHA diversity is generated through engineering the three basic synthesis pathways as well as PHA synthase specificity.

PHA biosynthesis contains three primary synthesis pathways, including the acetoacetyl-CoA pathway (pathway I), in situ fatty acid synthesis (pathway II), and/or beta-oxidation cycles (pathway III). PHA diversity is generated by engineering these pathways as well as utilizing PHA synthase specificity to producing specific polymers [99] (Figure 4). The structure of PHAs produced by the biosynthesis pathways mentioned above is not homogenous, as it is challenging to control homopolymers, random copolymers, and the ratios of monomers in the copolymers. Tailoring PHA structures, i.e., introducing new structures to PHA molecules, is achieved by altering monomers’ structure, feed, or growth conditions during the production of PHA to suit specific applications. To make tailoring of PHA structures possible, synthetic biology methods have been developed to diversify the PHA structures into homo-, random, and block polymers with enhanced properties to better meet various application requirements [62]. For instance, weakening the β-oxidation cycle in *Pseudomonas entomophila* and *P. putida* has led to a controllable synthesis of various PHA structures, including monomer ratios in random and/or block copolymers when fatty acids were used as PHA precursors [100]. PHA site chain grafting results in an infinite variation of PHA where the introduction of functional groups into PHA polymer chains in predefined proportions, the bacteria take up the fatty acids containing the functional groups for the PHA synthesis [100]. The above technology would allow cost-efficient biodegradable bioplastic production from PHA.

PHAome is a recently coined term that describes the diversity of monomer arrangements; homo-, random and block copolymers’ length, order, and type; as well as molecular weights of different microbial PHAs. PHAome also reflects the range of PHAs’ molecular weights and monomer ratios that occur in bacterial cells at particular time points during growth. Different bacterial species have different PHAomes because they contain different synthases and unique substrate preferences, but it is possible to manipulate and precisely custom-design PHA polymers in a particular bacterial species, which in turn promotes the discovery of new PHA properties and applications [101]. This way, a suitable bacterial platform can be constructed in order to provide consistent PHA molecular structures.

Furthermore, gene editing systems such as CRISPRi have been introduced recently as a tool to target genes involved in the synthesis of PHA and are considered a promising industrial producer of PHA, under open and continuous fermentation process conditions. In particular, the CRISPRi system was used to target genes of *prpC* and *gltA* in *Halomonas* species TD01, channeling more substrates to PHBV and PHB synthesis, respectively. By repressing the gltA gene, CRISPRi enhanced the accumulation of PHB by approximately 8%, with 10.22 ± 0.25 g/L cell dry weight and 77.68 ± 3.75 weight percent of PHB in dry cell weight, demonstrating that *Halomonas* sp. TD01 is a promising industrial producer of PHA [102].

## 9. Genome Engineering Prospects

Although not yet employed for bioplastic polymers or their feedstocks, large-scale genome engineering technologies are promising and flexible approaches that can positively impact industrial-scale production biopolymers, such as PHA, PHB, and PHBV. For example, Multiplex Automated Genome Engineering (MAGE) is a genome engineering method that has been used to scarlessly modify *E. coli*’s genome. Through MAGE, it is possible to use libraries of synthetic oligonucleotides and incorporate them synchronously into multiple sites of the targeted genome. With the use of successive MAGE cycles, it has been shown that more variants than the actual size of the cell population can be generated [103]. Wang et al. [103] generated 15 billion genetic variants of *E. coli* cells by optimizing 24 genes simultaneously to maximize the production of the industrially important isoprenoid lycopene in the cells. The colonies were then isolated and screened for variants on selective agar plates. The highest lycopene yield was ~9000 ppm. (μg per g dry cell weight), with the best-optimized biosynthesis pathways being *dxs* and *idi,* which increased lycopene production by 390% [103]. MAGE has evolved into “coselection MAGE” (CoS-MAGE), with higher insertion efficiency which means multiple promoters can be inserted simultaneously into multiple genomic operons [104]. In this way, Wang et al. [104] generated oligo pools of various T7 promoters that targeted 12 sites associated with the biosynthesis of aromatic amino acids in *E. coli*. CoS-MAGE technology resulted in *E. coli* H33 and H76 mutant strains with more than a fourfold increase in indigo pigment production and >8.6 mg g−1 dry cell weight of indigo. Finally, MAGE has also been successfully applied in the eukaryotic model *Saccharomyces cerevisiae* through yeast oligo-mediated genome engineering (YOGE), using yeast strains that produce biobased chemicals in industrial production [105]. YOGE technology generates genetically diverse strains that can be screened for desirable phenotypes.

Another recent innovation in genome engineering and synthetic biology is the synthetic chromosome rearrangement and modification using *loxP*-mediated evolution (SCRaMbLE). This system has been applied to the synthetic yeast genome project, Sc2.0 [106], and facilitated chromosome rearrangements to produce diverse strains with multiple genotypes [107]. The goal was to reduce *S. cerevisiae*’s genome by removing the non-essential genes and to address evolutionary questions via “SCRaMbLEing”. It is an efficient mutagenesis method to generate complex genotypes and diverse phenotypes of the chosen organism [108]. As in MAGE, this technology allows large-scale genome modifications and easy enzyme screening to generate microbial strains. The use of SCRaMbLE resulted in a *S. cerevisiae* yYW0399 strain that produced 1.7 μg per mg (dry weight) β-carotene pigment with a 5.1-fold increase in yield versus the original strain [109]. Multiplex SCRaMbLE Iterative Cycling (MuSIC) was developed in order to increase the production of bio-based chemicals where carotenoids production in *S. cerevisiae* yJBD069 mutant strain was increased to 37.39 mg L^−1^, a 38.8-fold change through 5 iterative cycles of SCRaMbLE [110]. The mentioned methods have the potential to optimize bacteria and yeast strains beyond traditional mutagenesis methods for improved biopolymer production.

## 10. Concluding Remarks and Perspectives

In addition to the tremendous potential microalgae have in relation to numerous therapeutic properties such as anticancer, anti-inflammatory, antimicrobial, and antioxidant [111,112], algae are also used for the production of biofuels [113], attesting to their natural ability to generate biopolymer precursors. New nuclear transformation techniques have enabled transgene approaches in algae, which in turn may mitigate problems faced during plant transformation and transgenic plant health in PHB-producing plants [114]. Algal antibacterial effects against bacterial contamination have been studied [115], and these may lower the cost of production compared to the high cost of sterilization needed in bioplastics production regarding bacteria. Algae grown on simple media do not require arable land and do not compete with traditional crops for agricultural space; therefore, the production of PHA in algae has several advantages over plants and non-algal microorganisms, as the cost of producing PHB by bacterial fermentation is high [79]. With the cost of terrestrial plant maintenance rising along with the deforestation and consumption of fertile terrestrial lands, the production of bioplastics in plants and non-photosynthetic microbes is becoming less appealing. Nevertheless, the commercialization of PHA produced in bacteria is thus far the most advanced when compared to PHA in other organisms. Still, bacterial production of PHA is not as cost-effective as petroleum-derived plastics due to several factors, such as high energy demands for sterilization, production control and intensive aeration, and the need for feedstock [116]. In addition, the production cost is increased by the low substrate to PHA conversions, where substrates are directed toward other purposes [117,118]. Microorganisms have also shown slow growth; for example, studies have shown that the yield of PHA accumulation increased by changing the cell division pattern and cell morphology of *E. coli* [119]. The extraction and purification of PHA products prove to be complex [120]. As a result, its potential to replace the more cost-effective petroleum-derived plastics has been delayed. On the other hand, while algae-based plastics are currently in their infancy stage, they will likely find applications in a wide range of industries once they are commercialized. With the advancement in synthetic biology and the emergence of new technologies, bioplastics production in microalgae may emerge as a leading option, given the low levels of demand that these organisms have on environmental resources.

## Data Availability

Publicly available datasets were analyzed in this study. This data can be found here: https://www.google.com/ (accessed on 12 October 2022).
